# Validation of the CAMCOG‐DS‐II, a neuropsychological test battery for Alzheimer's disease in people with Down syndrome: A Horizon 21 European Down syndrome Consortium study

**DOI:** 10.1002/alz.70071

**Published:** 2025-03-27

**Authors:** Phoebe Ivain, Asaad Baksh, Fedal Saini, Mina Idris, Miren Tamayo‐Elizalde, Jasmine Wells, Bessy Benejam, Sandra Virginia Loosli, Katja Sandkühler, Elisabeth Wlasich, Olivia Wagemann, Johannes Levin, Diane Martet, Silvia Sacco, Ségolène Falquero, Manon Clert, Anne‐Sophie Rebillat, Wan Ming Khoo, Madelaine Amelia Smith, Jessica Beresford‐Webb, Shahid Zaman, María Carmona‐Iragui, Laura Videla, Juan Fortea, Ellen Melbye Langballe, Ingrid Tøndel Medbøen, Frode Kibsgaard Larsen, Eleni Baldimtsi, Raphaella Paradisi, Panagiotis Ntailakis, Magdalini Tsolaki, Georgia Papantoniou, Eimear McGlinchey, Mary McCarron, Seán Kennelly, André Strydom

**Affiliations:** ^1^ Institute of Psychiatry Psychology, and Neuroscience Department of Neurodevelopmental Sciences King's College London London UK; ^2^ Barcelona Down Medical Center Fundació Catalana Síndrome de Down Barcelona Spain; ^3^ Department of Neurology LMU University Hospital LMU Munich München Germany; ^4^ Department of Neurology University Hospital Zurich Zurich Switzerland; ^5^ Institut Jêrome Lejeune Paris France; ^6^ Department of Psychiatry Herchel Smith Bldg, Robinson Way University of Cambridge Cambridge UK; ^7^ Sant Pau Memory Unit Neurology Department Biomedical Research Institute Sant Pau Hospital de la Santa Creu i Sant Pau Barcelona Spain; ^8^ Norwegian National Centre for Ageing and Health Vestfold Hospital Trust Tønsberg Norway; ^9^ Department of Geriatric Medicine Oslo University Hospital Oslo Norway; ^10^ Institute of Clinical Medicine Faculty of Medicine University of Oslo Oslo Norway; ^11^ Department of Neurology School of Medicine Aristotle University of Thessaloniki Thessaloniki Greece; ^12^ Greek Association of Alzheimer's Disease and Related Disorders (GAADRD) Thessaloniki Greece; ^13^ Laboratory of Neurodegenerative Diseases Center for Interdisciplinary Research and Innovation (CIRI ‐ AUTh) Balkan Center Aristotle University of Thessaloniki Thessaloniki Greece; ^14^ Laboratory of Psychology Department of Early Childhood Education School of Education University of Ioannina Ioannina Greece; ^15^ Trinity Centre for Ageing and intellectual Disability Ground Floor, Chemistry Building Extension, Lincoln Gate Trinity College Dublin Dublin Ireland; ^16^ Global Brain Health Institute Trinity College Institute of Neuroscience, Lloyd Building Trinity College Dublin Dublin Ireland; ^17^ National Intellectual Disability Memory Service, Russell Building Tallaght University Hospital, Tallaght Dublin Ireland

**Keywords:** Alzheimer's disease, cognitive decline, cognitive testing, Down syndrome, trisomy 21

## Abstract

**INTRODUCTION:**

The Cambridge Cognitive Examination modified for use in people with Down syndrome (CAMCOG‐DS) is a sensitive cognitive test for Alzheimer's disease (AD)–related decline in people with DS, but needs updates for sensitivity, cultural adaptability, and additional memory/executive function items. This study aimed to develop and validate the CAMCOG‐DS‐II.

**METHODS:**

In this multi‐language, multi‐site study, the psychometric properties of the CAMCOG‐DS‐II were evaluated against previously validated measures in 223 participants (mean age: 40.18 years) with DS across seven countries.

**RESULTS:**

The CAMCOG‐DS‐II had a high completion rate, minimal floor/ceiling effects (compared to the modified Cued Recall Test, the CANTAB Paired Associates Learning, and the Purdue Pegboard), strong validity and reliability, and performance was unaffected by language across sites. It differentiated between those with/without AD and distinguished clinically rated cognitively stable and prodromal individuals.

**CONCLUSION:**

The CAMCOG‐DS‐II is a sensitive measure of cognitive performance in people with DS at risk of AD. Its cross‐language and site reliability support its potential use in AD–DS clinical trials.

**Highlights:**

- Developed and validated the Cambridge Cognitive Examination modified for use in people with Down syndrome (CAMCOG‐DS‐II) for Alzheimer's disease in Down syndrome.
- CAMCOG‐DS‐II shows increased sensitivity to Alzheimer's disease–related decline in Down syndrome.
- Improved applicability across an international and culturally diverse population.
- Differentiates Alzheimer's disease status: cognitively stable, prodromal, and clinical.

## 1 BACKGROUND

Down syndrome (DS) is the most common genetic cause of intellectual disability.^1^ People with DS exhibit a complex health profile, and DS is recognized as a genetic variant of early‐onset Alzheimer's disease (AD) due to the triplication of the amyloid precursor protein gene on chromosome 21.^2^, ^3^, ^4^ In asymptomatic (pre‐diagnosis) individuals with DS, AD neuropathology has been observed from approximately age 35 in adults with DS, but the average age of clinical AD diagnosis is typically later, between 51 and 56 years of ager.^2^, ^5^, ^6^, ^7^

Cognition can be challenging to assess in people with DS as tests developed for use in the general population do not differentiate well between individuals who score in the lower ranges. This can often lead to the presence of floor effects when used with people with intellectual disabilities.^8^ Furthermore, the cognitive profile of people with DS is heterogeneous, and more variable across the lifespan than in the general population.^9^ People with DS can experience difficulties in verbal working memory and expressive language compared to the neurotypically developing population. However, they may also have non‐verbal and implicit memory skills that remain on a typical development trajectory for mental age.^9^, ^10^ These differences require tests specifically developed for people with DS to assess for AD.

The earliest AD‐related cognitive declines observed in people with DS include memory and executive functioning (verbal fluency and planning), eventually affecting other cognitive domains as well as functional abilities.^11^, ^12^

The Cambridge Examination for Mental Disorders of Older People with Down Syndrome and Others with Intellectual Disabilities (CAMDEX‐DS), was validated by Ball et al., as a standardized assessment pack used in diagnosing AD in DS.^13^, ^14^ It consisted of a caregiver interview and a neuropsychological test battery known as the Cambridge Cognitive Examination modified for use in people with DS (CAMCOG‐DS). The CAMCOG‐DS battery included subtests assessing orientation, language, memory, attention and calculation, praxis, abstract thinking, and perception.^15^ The CAMCOG‐DS has since become one of the most used tests to examine AD in DS and has been validated for use in several languages including Spanish, Portuguese, and German, among others.^16^, ^17^, ^18^

The CAMCOG‐DS showed high construct validity with the Mini‐Mental State Examination (MMSE), and it could track decline in people with AD longitudinally.^19^, ^20^ Moreover, it was reported to be sensitive to differential performance in people with DS who were classified as cognitively stable, in the prodromal stage of AD, or those with an AD diagnosis, using both subtests and total scores.^21^ Although the CAMCOG‐DS was designed for use in detecting AD‐related decline, it was found to also be sensitive to age‐related decline associated with AD in DS and has been used more broadly in people with intellectual disabilities.^14^, ^22^

Although the CAMCOG‐DS represents a sensitive test of AD in people with DS, its first edition was published in 1999, with some items now outdated (e.g., pictorial material of old‐fashioned phones) or culturally specific (e.g., items requiring knowledge of the British monarchy).^19^ Furthermore, the memory domain includes only a few items, with insufficient emphasis on the executive functioning domain. Consequently, there was a need to update some subtests to be culturally non‐specific, remove items lacking in sensitivity to cognitive decline in DS, and incorporate more items measuring memory and executive function.

The aims of this study were to develop and validate an updated version of the CAMCOG‐DS—the CAMCOG‐DS‐II—and provide a comprehensive psychometric examination of its validity in a large, international DS cohort. We examined completion rates, floor and ceiling effects, construct validity, test–retest reliability, internal consistency reliability, and discriminant abilities to distinguish AD participants from cognitively stable participants, in comparison with other cognitive tests used in people with DS. The validation of the caregiver interview of the CAMDEX‐DS‐II will be published separately.

## 2 METHODS

### 2.1 Development of the CAMCOG‐DS‐II

The development of the CAMCOG‐DS‐II was influenced by the findings of Aschenbrenner et al., which recommended a cognitive battery and identified key areas of cognition sensitive to early decline in people with DS, specifically: memory, language/executive function, attention, orientation, and praxis.^8^ The CAMCOG‐DS originally included the following domains: orientation, language (comprehension, expression), memory (new learning, retrieval of remote and recent information, registration, intentional thinking), attention, praxis (ideomotor and ideational), and visual perception.

**RESEARCH IN CONTEXT**

1. **Systematic review**: The current literature highlights a significant gap in cognitive assessment tools for individuals with Down syndrome (DS) to evaluate decline across various cognitive domains related to Alzheimer's disease (AD). Many existing assessments lack the sensitivity and specificity needed for this heterogenous population, resulting in a shortage of suitable outcome measures for clinical trials involving people with DS and AD.
2. **Interpretation**: Few cognitive tests have been developed specifically for diagnosing AD in people with intellectual disability. The Cambridge Cognitive Examination modified for use in people with DS (CAMCOG‐DS) is the most widely used cognitive assessment tool in this context but requires updates to better capture AD‐related decline in people with DS and reduce cultural specificity. The CAMCOG‐DS‐II provides increased sensitivity to AD‐related cognitive decline and improved applicability across an international and culturally diverse population.
3. **Future directions**: The CAMCOG‐DS‐II is an advancement in cognitive assessments for individuals with DS. Future work should focus on utilizing it for international clinical trials of AD, and exploring the relationship between the CAMCOG‐DS‐II longitudinal changes in the performance and fluid biomarkers of AD to provide further insights into the relationship with neuropathology and AD evolution. Further validation may be required in people with more severe levels of intellectual disability.

Members of the Horizon 21 Consortium, experienced in administering the CAMCOG‐DS, identified problematic items within these domains that were outdated, culturally specific, or believed to be too difficult or ambiguous to understand for people with DS. An expert panel, comprising research psychologists, neuropsychologists, psychiatrists, a geriatrician, and neurologists from the Horizon 21 Consortium with extensive clinical and research expertise in AD in DS, made iterative changes to these items within each subdomain of the CAMCOG‐DS. Face validity was established through a series of in‐person workshops conducted over 18 months, supplemented by additional discussions resolved via email or virtual meetings.

In addition, to adequately assess the memory and executive functioning domains, subtests were considered for inclusion in the CAMCOG‐DS‐II, based on (1) suitability of use across languages and cultures, and (2) previously used or validated in the DS population. The final selection included the cancellation task, overlapping images task, prospective memory task, and Cats & Dogs Stroop task.

1. Cancellation task: an assessment of attention and concentration. Participants are asked to find specific target images embedded within a background of similar stimuli. The time taken to complete the tasks and the number of correctly and incorrectly marked items are recorded.
2. Overlapping images task: designed to evaluate visual perception, this task requires participants to identify individual images within an overlaid composite image. The number of correctly identified images is scored.
3. Prospective memory task: a single‐trial assessment that evaluates prospective memory as a subdomain of executive function. The administrator informs the participant that an object (e.g., keys) will be hidden, and when an alarm sounds, they must remind the administrator to retrieve the object. Scoring reflects the participant's ability to remember the item and its location, categorized as spontaneous recollection, recollection requiring a prompt, recollection requiring a clue, or failure to remember.
4. Cats & Dogs Stroop task: this test measures inhibition through two trials. In this first trial, the participant is first asked to name each image (either a “cat” or “dog”) as quickly as possible. In the second part, they are instructed to provide the opposite name for each item, calling “cats” as “dogs” and vice versa. The number of correctly named images and the time to complete both trials are recorded.

Items such as memory (retrieval of remote and recent information) and praxis (ideational) were removed. Memory items were removed due to pronounced floor effects for people with all levels of intellectual disability and were replaced with the prospective memory task. The praxis items were replaced with the optional “Brief Praxis Test” provided in the supplementary material of the CAMCOG‐DS‐II. This change was made as the original praxis items had limited utility for assessing ideational apraxia, which typically occurs later in AD progression. A summary of these changes is provided in Table S1.

Following these updates, the CAMCOG‐DS‐II was translated into Spanish, French, Dutch, German, Norwegian, and Greek using a back‐translation process. Although it was translated into Dutch, the CAMCOG‐DS‐II was not administered to a Dutch cohort in this study. The initial translation was typically undertaken by members of the relevant site, agreeing wording with local experts, and a final consensus version was then back‐translated into English by one or two independent translators, after which any discrepancies were checked and resolved. Upon finalization of the translated versions, each site conducted a pilot testing phase over the course of a few months to ensure that there were no issues with the additional subtests and adapted items; minor changes and clarifications were made following this phase. A manual and training materials were then developed to standardize the administration and scoring of the CAMCOG‐DS‐II. All raters underwent training before implementing the CAMCOG‐DS‐II in their service.

### 2.2 Study design and participants

The Horizon 21 study is an observational, multi‐center, longitudinal study of AD in people with DS across seven countries (United Kingdom: London and Cambridge, France, Norway, Germany, Spain, Ireland, and Greece) and six different languages. For the CAMCOG‐DS‐II validation study, 223 adults with DS were recruited between March 2018 and March 2024 for baseline and test–retest examinations. Participants’ site and demographics information are listed in Table 1, with additional information in Table S2. Participants were categorized into two groups: younger adults (≤40 years of age), and older adults >40 years of age with and without AD (including those with prodromal symptoms if not diagnosed with AD). The rationale for this age division is based on the observed presence of AD histological features in people with DS from ≈30 years of age and older, and the likelihood of development of clinical symptoms after age 40.^7^ For the purposes of validation of the CAMDEX‐DS‐II, AD diagnosis was determined by the participants’ previous medical history, rather than based on the cognitive assessments performed as part of this study, although we also report CAMCOG‐DS‐II scores based on post‐test classification. For further details refer to Section 2.4.

**TABLE 1:** Participants demographics for the sample (%).

Total sample	223
Mean age at assessment (SD)	40.18 (11.45)
Age group	
Younger age ≤40	123 (55.2)
Older age >40	100 (44.8)
Level of intellectual disability^a^	
Mild	100 (44.8)
Moderate or severe	123 (55.2)
Sex	
Female	105 (47.1)
Male	118 (52.9)
AD diagnosis	
No	197 (88.3)
Yes	26 (11.7)
Mean age at AD diagnosis (SD)	50.72 (7.63)
Mean age at AD symptom onset (SD)	47.58 (7.80)
Site, *n* (% of total sample)	
Spain–Barcelona	26 (11.7)
U.K.–Cambridge	7 (3.1)
Ireland–Dublin	33 (14.8)
Greece	49 (22.0)
U.K.–London	36 (16.1)
Germany–Munich	31 (13.9)
Norway	21 (9.4)
France–Paris	20 (9.0)
Primary language	
English	76 (34.1)
French	20 (9.0)
German	31 (13.9)
Greek	49 (22.0)
Norwegian	21 (9.4)
Spanish	26 (11.7)

^a^ According to the ICD‐10.

#### 2.2.1 Inclusion criteria

All participants were 18 years of age and older and had a documented diagnosis of DS, as reported in their medical history. The capacity to consent was evaluated for participants at each assessment, and written, informed consent was obtained from all participants prior to inclusion. If there were any questions or concerns about the participants’ ability to consent, assent was sought from their personal appointee (family member/guardian/caregiver/support worker) in attendance and according to local regulatory requirements. This study was performed in accordance with the Declaration of Helsinki, and all data were anonymized according to good clinical practice guidelines and general data protection regulations prior to analysis. All procedures involving human subjects/patients/participants were approved by local ethic review boards (Table S3).

Participants were also required to be able to engage in cognitive assessments and be native or bilingual speakers so that language ability did not affect task performance. Caregivers were asked about the participant's willingness and ability to participate in tabletop or computer games. If caregivers deemed the participant able, they were invited to attend the study visit. Brief clinical screening for participant's visual and hearing abilities was done prior to task administration. Sufficient vision and hearing with or without correction to engage in table‐top cognitive assessments and to hear a loud conversation were required. In addition, to prevent learning effects, participants should have not been administered any of the same tests within the previous 6 months.

#### 2.2.2 Exclusion criteria

Individuals were not included in the study if they could not provide consent or if no assent was available. Participants were excluded if they were unable to engage in a basic cognitive assessment. Individuals with acute health problems (such as untreated mental health concerns, e.g., depression and anxiety) that could impact participation were also excluded. However, those with long‐term conditions who were stable and receiving treatment were not excluded.

### 2.3 Neuropsychological tests

The study assessment included the following measures: the CAMCOG‐DS‐II, and to assess construct validity, we used the CANTAB Paired Associates Learning (PAL),^23^ the modified Cued Recall Test (mCRT),^24^ and the Purdue Pegboard test scores.^25^

Each site counterbalanced the sequence of tests within the younger and older age groups with half of the participants starting with the CAMCOG‐DS‐II. All measures were collected within 1 month of the initial visit. Thirty‐one participants across all sites were invited to return for a test–retest of the CAMCOG‐DS‐II between 6 and 8 weeks following the initial assessment. Although the original study design intended to randomly approach one in four participants, randomization was often not feasible due to travel constraints, distance from the testing center, and scheduling conflicts within the 6‐ to 8‐week window.

To ensure consistency in test administration, in‐person training workshops were conducted for all cognitive raters. In addition, training videos and an assessment manual were developed and distributed to all sites. Most cognitive raters had prior experience with test administration; however, any new raters underwent in‐person training at the site level and were provided with the same training materials.

#### 2.3.1 CAMCOG‐DS‐II

The cognitive assessment of the updated CAMDEX‐DS, known as the CAMCOG‐DS‐II, is a neuropsychological test battery that assesses the following cognitive subdomains: orientation, language (expression and comprehension), memory‐new learning, praxis (drawing/copying, actions to command), perception, and executive function (fluency, attention, abstract thinking, prospective memory, and inhibition). Each subdomain receives an individual score, which are totaled together for a maximum score of 106. The higher the score, the better the cognitive ability. The range in subscale scores is shown in Table 2. Administration of the test takes ≈30 to 45 min, depending on participants’ needs, abilities, level of intellectual disability, and engagement with the tasks.

**TABLE 2:** Descriptive statistics of CAMCOG‐DS‐II scores: Mean, median, floor, and ceiling effects for all participants.

			Floor and ceiling effects				
					Count of participants scoring minimum and maximum scores (% of sample who completed test)		
Cognitive tests	Mean (SD)	Median (IQR)	Test minimum–maximum scores	Participant minimum–maximum scores	Minimum score	Maximum score	Count of participants who completed test
*CAMCOG‐DS‐II subtests*							
Orientation	8.64 (3.46)	10 (5–12)	0–12	0–12	2 (0.93)	61 (28.24)	216/223
Language	17.33 (4.95)	19 (14–21)	0–24	2–24	0 (0.00)	9 (4.17)	216/223
Memory – New Learning	11.35 (4.87)	13 (7–15)	0–21	0–20	1 (0.46)	2 (0.93)	216/223
Praxis	9.73 (4.28)	10 (6.5–13)	0–17	0–17	3 (1.39)	3 (1.39)	216/223
Perception	3.73 (1.44)	4 (3–5)	0–5	0–5	10 (4.67)	86 (40.19)	214/223
Executive function	14.88 (7.07)	16 (9–21)	0–27	0–27	3 (1.39)	2 (0.93)	216/223
CAMCOG‐DS‐II total score	65.64 (22.73)	70.5 (49.63–83)	0–106	8–101	0 (0.00)	0 (0.00)	216/223
*Comparator cognitive tests*							
PAL first trial memory score	9.04 (5.43)	10 (4–13)	0–26	0–20	7 (6.09)	0 (0.00)	115/202^a^
mCRT total immediate recall score	30.17 (8.27)	34 (28–36)	0–36	0–36	2 (1.15)	48 (27.59)	174/223
mCRT total delayed recall score	9.41 (3.70)	11 (9–12)	0–12	0–12	9 (5.29)	76 (44.71)	170/223
Purdue Pegboard total score	23.01 (11.36)	21 (15–32)	0–75	0–52	1 (1.23)	0 (0.00)	81/102^b^

^a^ Norway did not administer the CANTAB Paired Associates Learning task, hence the lower count of participant completion.

^b^ Greece, Germany, France, and Norway did not administer the Purdue Pegboard test, hence the lower count of participant completion.

#### 2.3.2 CANTAB PAL

The CANTAB PAL measures visuospatial short‐term memory from CANTAB (2016).^23^ Participants are asked to remember the location of patterns shown within boxes on a screen. The number of patterns shown increases at each level. The test is terminated if there are 10 failed attempts on any one level. The main outcome of the test is the PAL first trial memory score, reflecting the number of patterns remembered on the first try of each level. The administration time ranged from ≈10 to 20 min, depending on participant performance, as test duration is influenced by the number of attempts required to complete each level.

#### 2.3.3 mCRT

The mCRT is a task used to evaluate visuo‐verbal episodic memory in people with DS.^24^ This task includes a learning phase of 12 items of differing semantic categories presented on three cards. Participants are given a distinct cue for each item and asked to recall all four items on each card with a maximum of three attempts. A testing phase is then conducted with the participant asked to recall all 12 items across three trials of free and cued recall. A delayed recall trial is conducted after 20 min. The testing phase generates two measures: a free total immediate recall score (the sum of the spontaneous recall of the list of 12 items for each trial) and a total immediate score (spontaneous plus cued recall items). There are two additional outcome measures of the delayed recall trial: free delayed recall score (spontaneous items only) and total delayed recall score (spontaneous plus cued recall items). The total number of intrusion errors, or responses not corresponding to any of the items, during both free and cued recall for all trials is also recorded. Higher scores across immediate and delayed recall trials with fewer intrusions indicate better visuo‐verbal episodic memory. Administering all trials of the mCRT, including the delayed recall, takes ≈20 min.

#### 2.3.4 Purdue Pegboard test

The Purdue Pegboard test is a measure of praxis, coordination, and dexterity.^25^ The pegboard has two rows of 25 vertically aligned holes. The participant is asked to place as many pegs into the holes as they can in 30 s in three trials—using each hand individually and then both hands together. The total score is the number of pegs placed in holes during the time frame for each hand in addition to the score when using both hands together. A high number of pegs placed correctly within the allotted time and consistency between hands suggest better dexterity and fine motor coordination.

### 2.4 AD classification

Participants were initially grouped by whether they had received a clinical AD diagnosis prior to the assessment, which was reported in the medical history. To assess the accuracy of cognitive status classification based on the CAMDEX‐DS‐II caregiver interviews and diagnostic criteria, clinical raters from each site categorized participants after the assessment into one of the following groups: cognitively stable participants, those with prodromal AD, those with a diagnosis of AD, and those with an uncertain profile (cognitive decline due to identifiable non‐neurodegenerative causes). Clinicians conducted the classifications according to information provided in the assessment medical history, the CAMDEX‐DS‐II diagnostic criteria, the Diagnostic and Statistical Manual of Mental Disorders, Fifth Edition (DSM‐5) mild/major cognitive impairment criteria and mild/major cognitive impairment due to AD, and the International Statistical Classification of Diseases and Related Health Problems, 11th Revision (ICD‐11) dementia diagnostic criteria. Group performance was initially assessed on the CAMCOG‐DS‐II and comparator tests using clinical AD diagnosis (Table 3), and later using cognitive status classifications made by clinical raters following the assessment as a secondary measure of cognitive performance (Table 4).

**TABLE 3:** AD diagnosis group comparisons with demographics and performance on the CAMCOG‐DS‐II and comparator cognitive measures.

	AD Diagnosis		
	No	Yes	
*n*	197	26	*p‐*value
Age at assessment, mean (SD)	38.55 (10.91)	52.54 (7.11)	<0.001
Age group (%)			<0.001
Younger aged ≤ 40	122 (61.9)	1 (3.8)	
Older aged > 40	75 (38.1)	25 (96.2)	
Level of intellectual disability (%)			0.03
Mild	94 (47.7)	6 (23.1)	
Moderate or severe	103 (52.3)	20 (76.9)	
Site (%)^a^			0.264
Sex (%)			0.345
Female	90 (45.7)	15 (57.7)	
Male	107 (54.3)	11 (42.3)	
AD diagnosis (%)			<0.001
No	197 (100.0)	0 (0.0)	
Yes	0 (0.0)	26 (100.0)	
Age at dementia diagnosis, mean (SD)		50.72 (7.63)	NA
Age at dementia symptoms, mean (SD)		47.58 (7.80)	NA
**Orientation**			
Total score, median [IQR]	10.00 [7.00, 12.00]	4.00 [3.00, 6.50]	<0.001
**Language**			
Comprehension, median [IQR]	7.00 [5.00, 8.00]	4.00 [2.75, 7.00]	0.002
Expression, median [IQR]	12.00 [10.00, 14.00]	9.50 [6.75, 12.00]	0.001
Total score, median [IQR]	19.00 [15.00, 21.00]	14.50 [9.00, 18.25]	0.001
**Memory – New learning**			
Total score, median [IQR]	13.00 [7.00, 16.00]	7.00 [4.00, 9.25]	<0.001
**Praxis**			
Actions to command, median [IQR]	4.00 [3.00, 5.00]	4.00 [1.00, 4.25]	0.048
Drawing/copying, median [IQR]	6.25 [3.50, 9.00]	2.75 [0.88, 5.88]	<0.001
Total score, median [IQR]	10.50 [7.00, 13.50]	7.00 [2.38, 10.62]	0.001
**Perception**			
Total score, median [IQR]	4.00 [3.00, 5.00]	3.00 [1.00, 4.00]	<0.001
**Executive Function**			
Verbal fluency, median [IQR]	3.00 [2.00, 3.00]	1.00 [1.00, 2.50]	0.001
Prospective memory, median [IQR]	2.00 [1.00, 3.00]	1.00 [0.00, 2.00]	0.007
Inhibition, median [IQR]	4.00 [1.00, 5.00]	1.50 [0.00, 4.00]	0.01
Attention, median [IQR]	6.00 [4.00, 7.00]	3.00 [2.00, 5.00]	<0.001
Abstract thinking, median [IQR]	2.00 [0.00, 5.00]	0.00 [0.00, 2.00]	0.002
Total score, median [IQR]	16.50 [10.00, 21.00]	7.00 [4.00, 14.25]	<0.001
**CAMCOG total score**, median [IQR]	72.00 [55.38, 84.50]	47.00 [25.88, 56.00]	<0.001
**CANTAB PAL**			
PAL first trial memory score, median [IQR]	11.00 [6.00, 13.00]	3.00 [1.00, 6.50]	<0.001
**mCRT**			
Immediate recall total score, median [IQR]	34.00 [30.00, 36.00]	20.00 [10.00, 29.00]	<0.001
Delayed recall total score, median [IQR]	11.00 [9.00, 12.00]	4.00 [1.50, 9.00]	<0.001
Intrusions immediate recall score, median [IQR]	1.00 [0.00, 4.00]	8.00 [2.50, 13.50]	0.002
Intrusions delayed recall score, median [IQR]	0.00 [0.00, 2.00]	5.00 [1.50, 6.50]	<0.001
**Purdue Pegboard**			
Total score, median [IQR]	21.50 [15.00, 33.25]	17.00 [11.25, 19.00]	0.022

^a^ Breakdown of AD status by site cannot be shown due to small cell size.

**TABLE 4:** Cognitive grouping based on the CAMDEX‐DS‐II.

	Cognitively stable	Prodromal AD	AD	Uncertain profile	*p*‐Value
*n*	152	19	39	12	
Mean age at assessment (SD)	36.32 (10.18)	36.47 (6.67)	52.87 (6.44)	53.00 (4.07)	<0.001
Age group (%)					<0.001
Younger age ≤40	108 (71.1)	14 (73.7)	1 (2.6)	0 (0.0)	
Older age >40	44 (28.9)	5 (26.3)	38 (97.4)	12 (100.0)	
Level of intellectual disability (%)					0.093
Mild	75 (49.3)	9 (47.4)	11 (28.2)	4 (33.3)	
Moderate or severe	77 (50.7)	10 (52.6)	28 (71.8)	8 (66.7)	
Sex (%)					0.45
Female	67 (44.1)	9 (47.4)	20 (51.3)	8 (66.7)	
Male	85 (55.9)	10 (52.6)	19 (48.7)	4 (33.3)	
AD diagnosis (%)					<0.001
No	152 (100.0)	19 (100.0)	13 (33.3)	12 (100.0)	
Yes	0 (0.0)	0 (0.0)	26 (66.7)	0 (0.0)	
Mean age at AD diagnosis (SD)			50.72 (7.63)		
Mean age at AD symptoms (SD)			47.58 (7.80)		
*Median scores (IQR) on CAMCOG‐DS‐II subtests*					
Orientation, median [IQR]	11.00 [9.00, 12.00]	7.00 [5.00, 11.50]	4.00 [3.00, 6.00]	8.50 [5.00, 10.00]	<0.001
Language, median [IQR]	20.00 [16.00, 22.00]	15.00 [11.50, 18.00]	13.00 [10.00, 19.00]	16.00 [15.25, 20.75]	<0.001
Memory – New learning, median [IQR]	14.00 [10.00, 16.00]	10.00 [7.00, 13.00]	6.00 [4.00, 9.00]	7.50 [5.25, 10.75]	<0.001
Praxis, median [IQR]	11.50 [9.00, 14.00]	8.50 [6.25, 10.00]	6.00 [3.00, 8.50]	8.25 [4.25, 11.50]	<0.001
Perception, median [IQR]	5.00 [4.00, 5.00]	4.00 [3.00, 4.00]	3.00 [1.00, 4.00]	3.50 [1.25, 4.00]	<0.001
Executive function, median [IQR]	18.00 [13.00, 22.00]	14.00 [9.50, 15.50]	7.00 [3.00, 14.00]	14.00 [8.25, 19.75]	<0.001
CAMCOG‐DS‐II total score, median [IQR]	76.50 [63.50, 88.50]	55.50 [47.75, 64.50]	46.00 [26.00, 55.50]	66.00 [39.75, 70.50]	<0.001
*Median scores (IQR) on the comparator cognitive tests*					
PAL first trial memory score, median [IQR]	12.00 [8.00, 14.00]	2.00 [0.50, 3.00]	2.50 [1.00, 6.25]	8.00 [5.50, 9.50]	<0.001
mCRT total immediate recall score, median [IQR]	35.00 [32.00, 36.00]	30.00 [26.00, 33.00]	17.50 [11.00, 26.00]	35.00 [8.00, 35.50]	<0.001
mCRT total delayed recall score, median [IQR]	12.00 [10.00, 12.00]	9.00 [4.50, 11.50]	4.00 [1.75, 8.00]	9.00 [0.50, 11.50]	<0.001
Purdue Pegboard total score, median [IQR]	22.00 [15.50, 34.00]	11.00 [11.00, 11.00]	17.00 [11.25, 19.00]	20.00 [18.00, 23.50]	0.055

### 2.5 Statistical analyses

To validate the CAMCOG‐DS‐II as a measure of cognition in people with DS, a comprehensive psychometric evaluation was conducted. Partial and full completion rates for the CAMCOG‐DS‐II were first examined. To be included in this analysis, participants were required to have completed 80% of the CAMCOG‐DS‐II items. Floor and ceiling effects were examined by calculating the percentage of participants who scored at the minimum and maximum scores on CAMCOG‐DS‐II and the comparator cognitive tests. Floor effects occur when a substantial proportion of participants score the lower possible level, implying that the test may be too difficult to capture the full range in abilities or responses of its target population. Conversely, ceiling effects occur when a significant number of participants score at the highest possible level, suggesting that the test may be too easy and fail to capture the full range of expected responses. Construct validity of the CAMCOG‐DS‐II subtests was examined by correlating performance on comparator tests assessing similar domains. The memory subtest score and the CAMCOG‐DS‐II total score were correlated with the CANTAB PAL and the mCRT scores. The CAMCOG‐DS‐II praxis subtest and the total score were correlated with the Purdue Pegboard total score. These analyses used Spearman's correlation as these data were non‐normally distributed.

Test–retest reliability was investigated by calculating the intra‐class correlation coefficient (ICC) with 95% confidence intervals (95% CI) for 31 participants using the CAMCOG‐DS‐II total score. For this analysis, the two‐way random effects models were used, and the “single rater” unit was reported. Internal consistency reliability was analyzed using Cronbach's alpha with 95% CIs for the CAMCOG‐DS‐II subtests and total score. Furthermore, receiver‐operating characteristic (ROC) curve analyses were used for CAMCOG‐DS‐II subtests, total score, and the comparator cognitive tests to investigate their discriminant abilities to distinguish AD participants from cognitively stable participants, reporting the area under the curve (AUC) values with 95% CIs.

Group differences in performance were examined on the CAMCOG‐DS‐II and comparator cognitive tests for age group (younger: ≤40 years of age and older: >40 years of age), sex, level of intellectual disability, and AD diagnosis (clinician‐made diagnosis made prior to assessment) using the Mann‐Whitney *U* test for non‐parametric comparisons. We used descriptive statistics to compare CAMCOG‐DS‐II scores by AD status based on clinical ratings following the assessment. Next, demographic factors that might impact performance were explored using adjusted linear regression models, reporting the beta‐coefficient (*β*) with 95% CIs. This approach allowed us to evaluate the influence of individual factors (e.g., AD diagnosis) on performance, independently of the other variables in the model (e.g., age, sex, or level of intellectual disability).

Given the variation in languages across sites, separate linear regression models were conducted to estimate the effect of language on performance. In this sub‐analysis, only the younger participants were included, those without an AD diagnosis, and with mild intellectual disability to ensure equivalence in sample characteristics between language groups, due to the strong associations between these variables and cognitive performance. These models were adjusted for age and sex. English was the reference group for language, as this was the largest language group. All data analyses were conducted using R version 4.4.0.^26^

## 3 RESULTS

Due to a limited number of people with severe intellectual disability across the sites (Table S2, 4.9% of sample), moderate and severe intellectual disability groups were combined into a “moderate or severe group” for subsequent analyses. The level of intellectual disability was classified according to the ICD‐10 at all sites.^27^

The most common primary language of participants was English (34%). The mean age of participants was 40.18 years of age (standard deviation [SD] = 11.45), with 45% of participants being >40 years of age. There were marginally more male (53%) than female (47%) participants, and more participants with a moderate or severe level of intellectual disability (55%). A total of 26 participants (12%) had an AD diagnosis at the time of their assessment. Mean age at AD symptom onset was 47.58 years (SD 7.80 years) and mean age at AD diagnosis was 50.72 years (SD 7.63 years).

### 3.1 Psychometrics of the CAMCOG‐DS‐II and the comparator cognitive tests

#### 3.1.1 Completion rates and floor and ceiling effects

The overall combined full completion and partial‐completion rate (80% completion) for the CAMCOG‐DS‐II was 96.86% (216/223 participants; Table 2). Six participants could not complete any of the CAMCOG‐DS‐II subtests and one participant could complete only two subtests. Of the six participants who did not complete any of the CAMCOG‐DS‐II, one had mild intellectual disability, was cognitively stable, and cited a lack of motivation (although they completed the mCRT). Four had moderate intellectual disability: one was cognitively stable but unwilling to participate in tasks (although they completed the CANTAB PAL), one had prodromal AD and was unable to perform, and two had dementia and could not follow instructions (although one completed the Purdue Pegboard task). The sixth participant had severe intellectual disability, prodromal AD, and could not follow instructions. The participants who completed only two subtests had moderate disability, were cognitively stable, and they were unable to perform most tasks but completed the CANTAB PAL.

Across the six subtests of the CAMCOG‐DS‐II, floor effects were minimal (Table 2). Floor effects were present in 5% of participants who attempted the perception subtest. This subtest also exhibited the highest ceiling effect, with 40% of participants scoring the maximum score. In addition, 28% of participants obtained the maximum score on the orientation subtest. Overall, there were no floor or ceiling effects for the CAMCOG‐DS‐II total score. Floor and ceiling effects for the CAMCOG‐DS‐II subtests can be found in Table 2.

Floor effects were present in 6% of participants who completed the CANTAB PAL and 5% of participants who completed the mCRT for the immediate recall subtest. Ceiling effects were common for the mCRT: 28% for the total immediate recall score and 45% for the total delayed recall score. There were no ceiling effects for the CANTAB PAL or the Purdue Pegboard test.

#### 3.1.2 Construct validity

Performance on the CAMCOG‐DS‐II memory subtest correlated positively with the CANTAB PAL first trial memory scores (*r_s_* = 0.43, *p* < 0.001) and the mCRT total immediate recall scores (*r_s_* = 0.63, *p* < 0.001) and total delayed recall scores (*r_s_* = 0.57, *p* < 0.001). The CAMCOG‐DS‐II total praxis subtest scores correlated positively with the Purdue Pegboard total score (*r_s_* = 0.49, *p* < 0.001).

CAMCOG‐DS‐II total score correlated significantly with CANTAB PAL first trial memory scores (*r_s_* = 0.48, *p* < 0.001) and the mCRT total immediate recall scores (*r_s_* = 0.64, *p* < 0.001) and the total delayed recall scores (*r_s_* = 0.59, *p* < 0.001), as well as with the Purdue Pegboard total score (*r_s_* = 0.46, *p* < 0.001).

#### 3.1.3 Test–retest reliability

Each site conducted at least one test–retest visit. The mean age of participants who completed test–retest was 38.93 (SD = 10.54; range = 24–61 years of age). Twelve had mild and 19 had moderate intellectual disability. Twenty‐six participants were cognitively stable, two had a clinical AD diagnosis, one had prodromal AD, and two had an uncertain profile. Test–retest reliability was found to be good for most of the subtests of the CAMCOG‐DS‐II and total score and was within the commonly accepted range of 0.75–0.90. The CAMCOG‐DS‐II total score had an ICC = 0.83 (95% CI 0.72–0.91, *p* < 0.001). CAMCOG‐DS‐II subtest test–retest reliability was ICC = 0.81 (95% CI 0.67–0.89, *p* < 0.001) for orientation, ICC = 0.77 (95% CI 0.61– 0.87, *p* < 0.001) for language, ICC = 0.44 (95% CI 0.17–0.65, *p* = 0.006) for memory—new learning, ICC = 0.81 (95% CI 0.68–0.89, *p<* 0.001) for praxis, ICC = 0.87 (95% CI 0.75–0.93, *p* < 0.001) for executive function, and ICC = 0.49 (95% CI 0.23–0.68, *p* = 0.002) for perception.

#### 3.1.4 Internal consistency reliability

Internal consistency was calculated for each of the CAMCOG‐DS‐II subtests using Cronbach's alpha. Internal consistency was acceptable (0.7 ≤ *α* < 0.8) or good (0.8 ≤ *α* < 0.9) for all subtests: 6 items of the orientation subset, *α* = 0.81 (95% CI 0.77–0.85); 12 items of the language subtest, *α* = 0.85 (95% CI 0.82–0.88); 6 items of the memory subtest, *α* = 0.80 (95% CI 0.75–0.84); 7 items of the praxis subtest, *α* = 0.77 (95% CI 0.71–0.81); and 10 items of the executive functions subtest, *α* = 0.86 (95% CI 0.83–0.89). Internal consistency could not be calculated for the perception subtest as it included only one item.

Internal consistency for the 42 CAMCOG‐DS‐II items as a test of general cognitive abilities in people with DS was found to be excellent, with *α* = 0.95 (95% CI 0.94–0.96).

#### 3.1.5 Test performance by clinical AD diagnosis

Participants with a clinical AD diagnosis prior to assessment performed significantly poorer than participants without a diagnosis of AD on all CAMCOG‐DS‐II subtests and had a lower median CAMCOG‐DS‐II total score (47.00 vs 72.00; *p *< .001). Participants with AD also performed poorer than those without AD on the CANTAB PAL, with lower first‐trial memory scores (3.00 vs 11.00). They also had lower median total immediate recall scores (20.00 vs 34.00; *p *< .001) and delayed recall scores (4.00 vs 11.00; *p *< .001) on the mCRT and made more intrusion errors during both immediate recall (8.00 vs 1.00; *p *= .002) and delayed recall (5.00 vs 0.00; *p *< .001). Finally, they also showed worse performance on the Purdue Pegboard test compared to participants without an AD diagnosis (17.00 vs 21.50; *p *= .022) (Table 3).

#### 3.1.6 Distinguishing AD participants from cognitively stable participants

ROC curve analysis classifying AD (according to clinical diagnosis) from participants without an AD diagnosis showed that the memory‐new learning subtest of the CAMCOG‐DS‐II (AUC value = 0.76, 95% CI 0.68–0.84) was the best at distinguishing AD, followed by orientation (AUC value = 0.75, 95% CI 0.65–0.86) (Figure S1; Table S4). The overall AUC value for the CAMCOG‐DS‐II total score was 0.75 (95% CI 0.66–0.84). The CAMCOG‐DS‐II, CANTAB PAL, and mCRT were comparable at classifying AD from those without an AD diagnosis (PAL AUC 0.79, 95% CI 0.70–0.88; mCRT total immediate recall AUC 0.82, 95% CI 0.72–0.92; and mCRT total delayed recall AUC 0.80, 95% CI 0.69–0.90). The Purdue Pegboard was not as good at classifying AD as the other cognitive tasks (AUC 0.69, 95% CI 0.56–0.83) (Figure S2; Table S5).

#### 3.1.7 AD status following assessment

The cognitive status of participants was rated by clinicians following the assessment according to the CAMDEX‐DS‐II diagnostic criteria described previously. The descriptive statistics can be found in Table 4. The cognitively stable group performed better than the prodromal AD, uncertain, and AD groups across all domains of the CAMCOG‐DS‐II and the other tests. For the mCRT, immediate recall performance was at ceiling for the cognitively stable group and the uncertain group (both with median scores of 35). Performance on the memory and perception subtests of the CAMCOG‐DS‐II (median [IQR]) for the uncertain group (7.50 [5.25, 10.75]; 3.50 [1.25, 4.00]) was poorer than the for prodromal AD group (10.00 [7.00, 13.00]; 4.00 [3.00, 4.00]) but both had comparable executive functioning abilities (14.00 [8.25, 19.75]; 14.00 [9.50, 15.50]). The CAMCOG‐DS‐II total scores for the AD groups 46.0 [26.0, 55.5]) and prodromal AD (55.50 [47.75, 64.50]) were lower than those for the cognitively stable (76.50 [63.5, 88.5]) and uncertain groups (66.0 [39.75, 70.5]).

**FIGURE 1:**
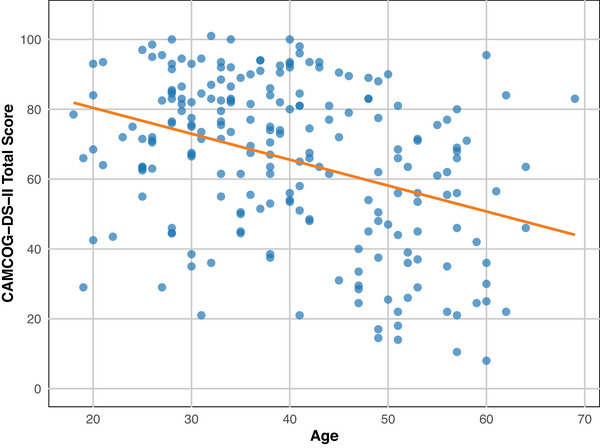
Relationship between CAMCOG‐DS‐II total score and age. CAMCOG‐DS‐II, Cambridge Cognitive Examination modified for use in people with Down syndrome.

Figure 1 demonstrates that with increasing age, performance on the CAMCOG‐DS‐II total score decreased. Exploring age group differences on each cognitive test showed statistically significant differences in performance in older (age >40) compared to younger (age ≤40) participants across all subtests and the total score of the CAMCOG‐DS‐II (Table S6). This was particularly evident for the CAMCOG‐DS‐II total score, showing a 19‐point difference in median scores between the two age groups (75.50 vs 56.50 points). Further group performance comparisons by age, sex, and level of intellectual disability can be found in Tables S6, S7, and S8.

#### 3.1.8 Factors associated with performance on CAMCOG‐DS‐II total score and comparator cognitive tests

Examining factors associated with performance on the CAMCOG‐DS‐II total score demonstrated that the older age group had significantly poorer scores compared to the younger while adjusting for sex, level of intellectual disability, and AD diagnosis (*β* = −12.44, 95% CI −18.01 to −6.86) (Table 5). People with moderate or severe intellectual disability performed poorer than those with mild intellectual disability (*β* = −17.59, 95% CI −22.81 to −12.38), as did people with an AD diagnosis compared to those without AD (*β* = −10.21, 95% CI −18.79 to −1.63). There were no significant differences in performance for CAMCOG−DS−II total score between males and females (*p* > .05).

**TABLE 5:** Factors associated with performance on the CAMCOG‐DS‐II and comparator cognitive tests.

	CAMCOG‐DS‐II total score		PAL first trial memory score		mCRT total immediate recall score		mCRT total delayed recall score		Purdue Pegboard total score	
Variable	*β* (95% CI)	*p*‐value	*β* (95% CI)	*p*‐value	*β* (95% CI)	*p*‐value	*β* (95% CI)	*p*‐value	*β* (95% CI)	*p*‐value
Age group										
Younger age ≤40	Ref.		Ref.		Ref.		Ref.		Ref.	
Older age >40	−12.44 (−18.01 to −6.86)	<0.001	−2.70 (−4.75 to −0.65)	0.01	−2.81 (−5.32 to −0.31)	0.03	−0.94 (−2.10 to 0.21)	0.11	−8.81 (−13.81 to −3.80)	<0.001
Sex										
Female	Ref.		Ref.		Ref.		Ref.		Ref.	
Male	−0.99 (−6.16 to 4.18)	0.71	0.66 (−1.26 to 2.58)	0.50	0.32 (−1.92 to 2.56)	0.78	0.23 (−0.81 to 1.26)	0.67	0.002 (−4.68 to 4.69)	1.00
Level of intellectual disability										
Mild	Ref.		Ref.		Ref.		Ref.		Ref.	
Moderate or severe	−17.59 (−22.81 to −12.38)	<0.001	−0.81 (−2.71 to 1.08)	0.40	−3.20 (−5.43 to −0.96)	0.005	1.62 (−2.66 to −0.59)	0.002	−4.05 (−8.65 to 0.55)	0.08
AD diagnosis										
No	Ref.		Ref.		Ref.		Ref.		Ref.	
Yes	−10.21 (−18.79 to −1.63)	0.02	−3.64 (−6.54 to −0.74)	0.01	−8.36 (−12.46 to −4.26)	<0.001	−3.23 (−5.10 to −1.35)	<0.001	−2.49 (−9.65 to 4.68)	0.49
**Model statistics**	*R* ^2^ = 0.31, *F* (4, 211) = 24.81, *p* < 0.001		*R* ^2^ = 0.15, *F* (4, 111) = 6.03, *p* < 0.001		*R* ^2^ = 0.19, *F* (4, 170) = 11.07, *p* < 0.001		*R* ^2^ = 15, *F* (4, 166) = 8.58, *p* < 0.001		*R* ^2^ = 0.18, *F* (4, 77) = 5.31, *p* < 0.001	

On the CANTAB PAL first trial memory score, the older group performed poorer than younger individuals (*β* = −2.70, 95% CI −4.75 to −0.65). Compared to people without AD, those with AD also performed poorer on the PAL (*β* = −3.64, 95% CI −6.54 to −0.74). Sex and level of intellectual disability were not associated with performance on the PAL first trial memory score in those who attempted the task (both *p* > .05).

On the mCRT total immediate score, the older group performed poorer than the younger group (*β* = −2.81, 95% CI −5.32 to −0.31), as did those with moderate or severe intellectual disabilities compared to mild intellectual disability (*β* = −3.20, 95% CI − 5.43 to −0.96). Moreover, having an AD diagnosis was associated with poorer performance (*β* = −8.36, 95% CI −12.46 to −4.26). On the mCRT delayed recall total score, only moderate or severe intellectual disabilities (*β* = 1.62, 95% CI −2.66 to −0.59) and an AD diagnosis (*β* = −3.23, 95% CI −5.10 to −1.35) were associated with poorer performance. Finally, when comparing total scores, the older group performed poorer than the younger on the Purdue Pegboard test (*β* = −8.81, 95% CI −13.81 to −3.80). Sex, level of intellectual disability, and AD diagnosis were not associated with performance on the Purdue Pegboard total score (all *p* > .05).

#### 3.1.9 Language‐dependent variations in CAMCOG‐DS‐II total score and comparator cognitive test performance

To examine the effect of language on the CAMCOG‐DS‐II total score, a regression analyses was conducted in younger participants without an AD diagnosis and with mild intellectual disability. After adjusting for age and sex, the model was not significant (*R* ^2^ = 0.09, *F* (7, 54) = 1.88, *p* = 0.09). Similarly, language did not have a statistically significant effect on the mCRT total immediate score (*R* ^2^ = 0.03, *F* (7, 52) = 1.30, *p* = 0.27), mCRT total delayed recall score (*R* ^2^ = 0.02, *F* (7, 51) = 1.14, *p* = 0.35), or the Purdue Pegboard total score (*R* ^2^ = 0.19, *F* (3,15) = 2.42, *p* = 0.11).

However, on the PAL first trial memory score, participants who spoke Greek performed poorer than English‐speaking participants (*β* = −6.42, −10.64 to −2.21, *p* = 0.004, *R* ^2^ = 0.38, *F* (6,26) = 4.30, *p* = 0.004).

## 4 DISCUSSION

This study sought to examine the psychometric properties of the CAMCOG‐DS‐II, an updated cognitive assessment tool for people with DS using a large international cohort. The CAMCOG‐DS‐II achieved a high completion rate of 96.86%, with the total score showing no floor or ceiling effects. The tool demonstrated good construct validity, which was specifically assessed for the memory and praxis scales, and strong internal consistency and test–retest reliability.

### 4.1 Diagnosing AD in people with DS

The present results indicate that the CAMCOG‐DS‐II is sensitive to AD‐related decline and can help distinguish participants with a clinical AD diagnosis from those without a diagnosis. Across total and subtest scores, people with DS and AD performed significantly poorer compared to participants without an AD diagnosis. The total score also exhibited a robust diagnostic classification of participants with AD compared to those without a diagnosis. When comparing the diagnostic classification of the CAMCOG‐DS‐II subtests, the memory‐new learning domain was the most sensitive to distinguishing participants with AD from those without a diagnosis, followed by the orientation and executive function subtests. This is concurrent with the previous literature, which has found memory and executive function to be the earliest cognitive domains to be affected by AD in people with DS.^11^

The CAMCOG‐DS‐II's sensitivity to differentiating individuals with AD from those without appears to be higher than for other tests that have previously been employed in clinical trials of AD in DS. For instance, clinical trial outcome measures such as the Brief Praxis Test^28^ and the Severe Impairment Battery^29^ show only modest AD discrimination in DS (AUC = 066; 0.61).^28^, ^30^, ^31^, ^32^, ^33^ In contrast, the CAMCOG‐DS‐II subtests and total scores showed higher sensitivity (all AUC's > 0.68). Therefore, the CAMCOG‐DS‐II may offer more valuable information for clinical trials for AD in DS. We acknowledge, as is common to all cognitive testing, that personal or motivational factors (e.g., mood, anxiety, or boredom) may affect performance, which may impact longitudinal variability for repeat assessments. This could be minimized by ensuring consistent testing circumstances and addressing any environmental factors that could impact performance. In this study, we have standardized assessments and excluded people who completed less than 80% of the CAMCOG‐DS‐II to optimize performance (*n* = 7).

In addition, the CAMCOG‐DS‐II demonstrated the ability to discern participant cognitive status. The CAMCOG‐DS‐II total scores showed a significant association with cognitive status as classified by clinical raters. Specifically, the CAMCOG‐DS‐II total scores differed between cognitively stable, prodromal AD, AD participants, and those with an uncertain cognitive profile, indicating that this test is sensitive to differing degrees of cognitive impairment. Of interest, the “uncertain” group's poorer performance on memory and perception subtests compared to the prodromal AD group highlights the need for nuanced interpretation of cognitive testing. Factors such as health comorbidities, physical health decline, sensory impairments, or significant life events leading to mental health challenges can all influence test performance. The present results suggest that the CAMCOG‐DS‐II may offer a more detailed evaluation of the cognitive status of people with DS, providing more information relevant to early intervention strategies and prodromal AD.

### 4.2 Floor and ceiling effects

The CAMCOG‐DS‐II total score showed no floor and ceiling effects. Most subtests had minor floor or ceiling effects, apart from the perception and orientation subtests, which had ceiling effects in 40% and 28% of participants, respectively. The ceiling effect observed in the perception subtest does not compromise the overall validity of the CAMCOG‐DS‐II, as perception is considered a less central domain for AD diagnosis in people with DS.^8^ Conversely, orientation is a critical domain for AD diagnosis. Despite the presence of ceiling effects, orientation showed high sensitivity in distinguishing participants with an AD diagnosis from those who were cognitively stable and outperforming other subtests in this regard. Both perception and orientation subtests assess clinical constructs and use binary or near‐binary scoring (e.g., correct, partially correct, incorrect, n/a), which can result in ceiling effects in cognitively stable populations, which comprised the majority of the cohort. Nevertheless, these subtests are valuable for assessing AD progression and conversion, as they are sensitive to AD‐related changes, even in individuals performing well at baseline.^8^ In comparison, the original CAMCOG‐DS total score reported floor effects for 11% of participants, with floor effects in subdomains ranging from 13.5% to as high as 78.4%.^19^ One goal of updating the CAMCOG‐DS was to ensure that it was appropriately feasible for participants, allowing the capture of relevant cognitive decline information across a broad spectrum of intellectual disability levels.

When compared to the other tests, ceiling effects were observed in both the mCRT total immediate and delayed recall scores, whereas such effects were minimal in the memory subtest of the CAMCOG‐DS‐II. This suggests that relative to its initial version and the other tests evaluated, the CAMCOG‐DS‐II is well calibrated for its intended participant base.

### 4.3 Factors associated with performance

When assessing factors influencing performance on the CAMCOG‐DS‐II, older adults, compared to the younger age group, had worse performance when adjusting for sex, level of intellectual disability, and clinical AD diagnosis. Previous research has identified a clear age effect on cognitive performance, emphasizing the relationship between older age and cognitive decline in the DS population.^34^, ^35^, ^36^ Age is also a significant predictor of AD in people with DS, with AD neuropathological features often observed by age 30 and early diagnoses typically occurring, with mean age of 50.72 in our cohort.^37^, ^38^ Therefore, it is crucial that the CAMCOG‐DS‐II effectively captures age‐related cognitive decline.

Previous studies have also reported declines in neuropsychological functions and behavioral skills in people with DS, occurring anywhere from 10 years to as much as 20–30 years before an AD diagnosis.^34^, ^39^ Specifically, significant cognitive declines in older participants, compared to younger ones, have been observed in executive functions, short‐term memory, and orientation.^34^, ^38^ These findings are consistent with the cognitive domains identified in our study as most sensitive for distinguishing participants with AD from those who are cognitively stable.

Another factor influencing performance on the CAMCOG‐DS‐II was the level of intellectual disability. As expected, participants with moderate or severe intellectual disability performed poorer than those with mild disability across all subtests and the total score. This indicates that the CAMCOG‐DS‐II is sensitive to varying levels of cognitive functioning and can be administered across a range of levels of intellectual disabilities, but also implies that scores will need to be interpreted according to the individual's level of baseline ability. Although people with severe intellectual disability comprised only 4.9% of the sample, it remains important to continue including this group in testing. Further exploration is needed to evaluate the suitability of the CAMCOG‐DS‐II for use in this population. Historically, cognitive tests for dementia‐related decline have been unavailable for individuals with severe intellectual disability.^40^, ^41^ Including this group in further validation studies is critical to ensuring whether tools can effectively diagnose AD across a widely heterogenous group with intellectual disability.

There was no effect of sex on cognitive performance on the CAMCOG‐DS‐II, which is consistent with the findings reported for the CAMCOG‐DS and prior cognitive tests conducted within DS cohorts.^19^, ^37^ Although differences have been reported in AD prevalence, clinical presentation, earliest affected cognitive domains, burden of neuropathology, and differential rates of cognitive decline observed between men and women in the general population, this has not yet been thoroughly explored and defined in people with DS.^42^

### 4.4 Language effects

The CAMCOG‐DS has been translated into several languages,^16^, ^17^, ^18^ but there is currently no research investigating how different languages may impact performance. In this study, which included data from six languages, no language effect was found on CAMCOG‐DS‐II performance among younger participants without an AD diagnosis and with mild intellectual disability. These findings suggest that the test performs consistently across languages in our cohort, highlighting its potential utility as an international tool for standardized, multilanguage studies on AD in DS. After decades of being overlooked as potential participants in clinical trials for AD, individuals with DS are now included increasingly in studies examining AD‐related interventions.^2^, ^43^ Consequently, there is a growing need for cognitive endpoints that are not influenced by language and can be applied in international clinical trials of AD in DS. Our findings suggest that the CAMCOG‐DS‐II, which has shown consistent performance across multiple languages and utility within a heterogenous population, could serve as a valuable cognitive endpoint for these trials.

### 4.5 Strengths and limitations

This study included a large sample size from an international DS consortium across a multi‐center and multi‐language population. Internationally validated measures of cognitive decline designed for people with DS can provide comparable outcome measures for use in clinical trials and studies for which previously, people with DS were excluded.

This study had several limitations. First, the sites differed in sample size, age range, and level of intellectual ability of participants included, thereby limiting the power of within‐group analyses. In addition, this analysis is cross‐sectional. We are continuing annual cognitive assessments, which will enable longitudinal validation of the CAMCOG‐DS‐II. Furthermore, differing national health systems may affect the age at which AD diagnoses are reported. We attempted to minimize these effects by standardizing an assessment protocol for all included sites and having monthly consortium meetings to discuss study progression and scoring.

Differences in site capacity also affected the administration of comparator tests, such as the CANTAB PAL and the Purdue Pegboard. Another limitation of this study was the relatively small number of participants with a diagnosis of AD at baseline (*n* = 26). Although this is a smaller subset compared to the overall study cohort, it is comparable to previous studies, such as the validation of the CAMCOG‐DS‐I, which included a total of 74 participants, 10 of whom met the clinical criteria for an AD diagnosis.^15^ This highlights the challenges of recruiting participants with AD to research studies, particularly in the context of DS, where barriers such as logistical burdens, scheduling difficulties, and limited trust or awareness of research benefits have been reported.^44^

## 5 FUTURE DIRECTIONS

Although further research is needed to confirm the longitudinal performance of the CAMCOG‐DS‐II, our results provide a promising foundation. Future work will aim to further validate the CAMCOG‐DS‐II in a longitudinal sample of individuals with DS at risk of developing AD. Further analyses will also investigate how CAMCOG‐DS‐II performance correlates with fluid biomarkers and AD‐related changes in DS, as well as compare performance against the original CAMCOG‐DS. In addition, work to establish a minimal clinically important difference is required.

## 6 CONCLUSIONS

The CAMCOG‐DS‐II has demonstrated strong psychometric properties as a cognitive assessment tool for people with DS. Its high completion rate, absence of floor and ceiling effects in the total score, robust construct validity and reliability, as well as sensitivity for the differentiation of those with and without AD, highlight its diagnostic utility. Furthermore, there was consistent performance across languages and sites, underscoring the potential of the CAMCOG‐DS‐II as an international tool for AD research, particularly in clinical trials where cognitive endpoints are needed in diverse populations. The CAMCOG‐DS‐II has the potential to show improved detection of AD‐related cognitive decline, which will provide valuable insight into the cognitive status of people with DS across all levels of intellectual disability.

## CONFLICT OF INTEREST STATEMENT

J. Fortea reports grants from the Fondo de Investigaciones Sanitario, Carlos III Health Institute (INT21/00073, PI20/01473, and PI23/01786) and the Centro de Investigación Biomédica en Red sobre Enfermedades Neurodegenerativas (CIBERNED) Program 1, partly jointly funded by Fondo Europeo de Desarrollo Regional, Unión Europea, Una manera de hacer Europa. This work was also supported by National Institutes of Health grants (1R01AG056850‐01A1; R21AG056974, R01AG061566,1R01AG081394‐01, and 1R61AG066543‐01 to J. Fortea), the Department de Salut de la Generalitat de Catalunya (SLT006/17/00119 to J. Fortea), and Fundación Tatiana Pérez de Guzmán el Bueno (IIBSP‐DOW‐2020‐151). It was also supported by Horizon 2020–Research and Innovation Framework Programme from the European Union (H2020‐SC1‐BHC‐2018‐2020 to J Fortea). J. Fortea reported receiving personal fees for service on the advisory boards, adjudication committees, or speaker honoraria from AC Immune, Adamed, Alzheon, Biogen, Eisai, Esteve, Fujirebio, Ionis, Laboratorios Carnot, Lilly, Life Molecular Imaging, Lundbeck, Perha, and Roche, outside the submitted work. J. Fortea reports holding a patent for markers of synaptopathy in neurodegenerative disease (licensed to Adx, EPI8382175.0). M. Carmona‐Iragui reports grants from the Fondo de Investigaciones Sanitario (FIS), Instituto de Salud Carlos III (PI18/00335, PI22/00758, ICI23/00032) and the CIBERNED program (Program 1, Alzheimer Disease and SIGNAL study, www.signalstudy.es), partly jointly funded by Fondo Europeo de Desarrollo Regional, Unión Europea, Una manera de hacer Europa; Alzheimer's Association (AARG‐22‐973966), Global Brain Health Institute (GBHI_ALZ‐18‐543740), Jérôme Lejeune Foundation (#1913 cycle 2019B), and Societat Catalana de Neurologia (SCN2020). M. Carmona‐Iragui reported receiving personal fees for service on the advisory boards, speaker honoraria, or educational activities from Esteve, Lilly, Neuraxpharm, and Roche. All other authors report no declarations of interest. Author disclosures are available in the Supporting Information.

## CONSENT STATEMENT

Written informed consent from all participants or their legally authorized representatives were obtained in all cohorts prior to inclusion.

## DIVERSITY, EQUITY, AND INCLUSION STATEMENT

This research study incorporated several steps to address diversity, equity, and inclusion in the study recruitment, design, and interpretation.

## STUDY DESIGN AND RECRUITMENT

For study design, each site implemented recruitment strategies to reach a diverse participant sample. These strategies included outreach to local health care and research centers, care homes, Down syndrome (DS) organizations, charities, and networks of participant identification centers. This study was open to adults with DS across all levels of intellectual disability, including those who could not consent for themselves. In cases where participants could not consent for themselves, assent was required from a legally authorized representative.

## ACCOMMODATIONS AND SUPPORT

To promote equitable participation, we provided accommodations tailored to participants’ needs. These included flexible study visit scheduling options, paid and organized transportation to and from visits, provision of paid and organized meals during visits, and adherence to culturally and religiously sensitive protocols. In addition, employed research staff were all trained in intellectual disability research practices.

## DATA INTERPRETATION

In this initial study cross‐sectional analysis, we did not explicitly account for sample diversity beyond examining the impact of language and sex on outcome measure performance. Other social determinants such as race, ethnicity, and socioeconomic status were not assessed directly as part of our presented findings due to small numbers when broken down by country. Future research will aim to explore these factors and any impact on outcomes.

## Supporting information



Supporting Information

Supporting Information
