# Supplementary material for: Validation of the CAMCOG‐DS‐II, a neuropsychological test battery for Alzheimer's disease in people with Down syndrome: A Horizon 21 European Down syndrome Consortium study
Source: Alzheimers Dement. 2025 Mar 27;21(3):e70071. doi: 10.1002/alz.70071 (PMC11947767; doi:10.1002/alz.70071)
Supplement: Supplementary file 2 — Supporting Information [file ALZ-21-e70071-s001.docx]

**SUPPLEMENTARY MATERIALS**

**Table S1. Summary of item changes made in the CAMCOG-DS-II**

| **CAMCOG-DS Item** | **CAMCOG-DS-II Item** |
| --- | --- |
| **Orientation** | |
| 172. What is your name? | 172. What is your full name? |
| 173. What day is it today?  *If no response ask: Is it ___, ___ or ___?*  (correct day of the week plus two others – correct answer 2^nd^) | 173. What day is it today?  *If no response ask: Is it ___, ___ or ___?*  (correct day of the week plus two others – correct answer 2^nd^) |
| 174. What month is it now?  *If no response ask: Is it ___, ___ or ___?*  (correct month plus two others – correct answer 1^st^) | 174. What month is it now?  *If no response ask: Is it ___, ___ or ___?*  (correct month plus the previous month and the following month - correct answer 3^rd^) |
| 175. What year is it now?  *If no response ask: Is it ___, ___ or ___?*  (correct year plus two others – correct answer 3^rd^) | 175. What year is it now?  *If no response ask: Is it ___, ___ or ___?*  (correct year plus the previous year and the following year – correct answer 2^nd^) |
| 176. What is the name of this place? (or if tested at home: What is this address?)  *If no response ask: Is it ___, ___ or ___?*  (correct place plus two alternatives – correct answer 2^nd^) | 176. What is the name of this place? (or if tested at home: What is this address?)  *If no response ask: Is it ___, ___ or ___?*  (correct place plus two alternatives – correct answer 2^nd^) |
| 177. What is the name of this town (village, city)?  *If no response ask: Is it ___, ___ or ___?*  (correct town plus two alternatives – correct answer 1^st^) | 177. What is the name of this town (village, city)?  *If no response ask: Is it ___, ___ or ___?*  (correct town plus two alternatives – correct answer 1^st^) |
| **Language – Comprehension** | |
| 178. Please nod your head | 178. Please nod your head |
| 179. Please touch your right ear with your left hand | 179. Please put this pencil on your lap and then place it back on the table |
| 180. Please look at the ceiling and then look at the floor | 180. Please look at the ceiling and then look at the floor |
| 181. Please tap each shoulder twice with two fingers | 181. Please tap each shoulder twice with two fingers |
| **Language – Expression** | |
| 182. What is this called (pencil)? What is this called (wristwatch)? | 182. What is this called (pencil)? What is this called (wristwatch)? |
| 183. I am going to show you some pictures. Please tell me the name of each one.   - Shoe - Computer - Scales - Suitcase - Clock - Lamp | 183. I am going to show you some pictures. Please tell me the name of each one.   - Shoe - Tree - Book - Suitcase - Clock - Lamp |
| 184. I’d like you to tell me as many different animals as you can. See how many you can think of in one minute. | 184. I’d like you to tell me as many different animals as you can. See how many you can think of in one minute. |
| 185. What do you do with a hammer?  *Scoring:*  *Any correct use: 1*  *Incorrect: 0*  *Not asked: 9* | 185. What do you do with a hammer?  *Scoring:*  *To hit (object): 2*  *To hit: 1*  *Incorrect: 0*  *Not asked: 9* |
| 186. Where do people usually go to buy medicine? | 186. Where do people usually go to buy medicine? |
| 187. What is a bridge? | 187. What is a coat? |
| 188. I am going to say something and I’d like you to repeat it after me: ‘People spend Money.’ | 188. I am going to say something and I’d like you to repeat it after me: ‘People spend Money.’ |
| **Memory – New Learning** | |
| 189. I showed you some pictures a little while ago. Can you remember what they were?   - Shoe - Computer - Scales - Suitcase - Clock - Lamp | 189. I showed you some pictures a little while ago. Can you remember what they were?   - Shoe - Tree - Book - Suitcase - Clock - Lamp |
| 190. Which one of these pictures did I show you before?   - Shoe - Computer - Scales - Suitcase - Clock - Lamp | 190. Which one of these pictures did I show you before?   - Shoe - Tree - Book - Suitcase - Clock - Lamp |
| **Memory – Retrieval of Remote Memories** | |
| 191. Who was John Lennon?  Clue: He was in a famous pop group | *Removed* |
| 192. Which Princess died in a car crash in Paris?  Clue: She was married to Prince Charles | *Removed* |
| **Memory – Retrieval of Recent Information** | |
| 193. Who is the Prime Minister?  Clue: His/her first name is … (give first name) | *Removed* |
| 194. What is the name of the present king or queen?  Clue: It begins with … (give first letter) | *Removed* |
| **Prospective Memory** | |
|  | 191. These are my keys. I’m going to put them somewhere safe. Can you remind me about my keys when the alarm sounds? |
| **Attention / Concentration** | |
| 195. I would like you to count to twenty for me. | 192. *Cancellation task* |
| 196. *Counting fingers.* | 193. *Cats and dogs task* |
| 197. I’m going to say some numbers and I’d like you to repeat them after me…  *Scoring:*  *4 or 5 digit series correct: 3*  *2 or 3 digit series correct: 2*  *1 digit repeated: 1*  *0 correct: 0*  *Not asked: 9* | 195. I’m going to say some numbers and I’d like you to repeat them after me…  *Scoring:*  *4 or 5 digit series correct: 4*  *3 digit series correct: 3*  *2 digit series correct: 2*  *1 digit repeated: 1*  *0 correct: 0*  *Not asked: 9* |
| **Language – Comprehension** | |
| 198. I would like you to read this and do what it says.  ‘Close your eyes’ | 195. I would like you to read this and do what it says.  ‘Close your eyes’ |
| 199. I would like you to read this and do what it says.  ‘Close your eyes’ | 196. I would like you to read this and do what it says.  ‘Close your eyes’ |
| **Praxis – Copying and Drawing** | |
| 200. Copy this shape (circle)  *Scoring:*  *Correct: 1*  *Incorrect: 0*  *Not asked: 9* | 197. Copy this shape (circle)  *Scoring:*  *Correct: 1*  *Partially correct: 0.5*  *Incorrect: 0*  *Not asked: 9* |
| 201. Copy this shape (square)  *Scoring:*  *Correct: 1*  *Incorrect: 0*  *Not asked: 9* | 198. Copy this shape (square)  *Scoring:*  *Correct: 1*  *Partially correct: 0.5*  *Incorrect: 0*  *Not asked: 9* |
| 202. Copy this picture (3D house)  *Scoring:*  Outline of house: __  Windows, doors and chimney in correct positions: __  3D representation: __  Total: [ ]  *Not asked: 9* | 199. Copy this picture (3D house)  *Scoring:*  Outline of house:  Unit correct: 1  Unit distorted, incomplete but recognisable: 0.5  Absent or unrecognisable: 0  Windows, doors and chimney in correct positions:  Unit correct, placed properly: 2  Unit correct, placed poorly: 1  United distorted, incomplete but recognisable, placed properly: 1  Unit distorted, incomplete but recognisable, placed poorly: 0.5  Absent or unrecognisable: 0  3D presentation:  Unit correct, placed properly: 2  Unit correct, placed poorly: 1  United distorted, incomplete but recognisable, placed properly: 1  Unit distorted, incomplete but recognisable, placed poorly: 0.5  Absent or unrecognisable: 0  Total: [ ]  *Not asked: 9* |
| 203. Draw a large clock and put all the numbers on it.  Now set the hands to 10 past 11.  *Scoring:*  Circle or square: __  All numbers in correct position: __  Correct time: __  Total: [ ]  *Not asked: 9* | 200. Draw a large clock and put all the numbers on it.  Now set the hands to 10 past 11.  *Scoring:*  Circle or square:  Unit correct: 1  Unit distorted, incomplete but recognisable: 0.5  Absent or unrecognisable: 0  All numbers in correct position:  Unit correct, placed properly: 2  Unit correct, placed poorly: 1  United distorted, incomplete but recognisable, placed properly: 1  Unit distorted, incomplete but recognisable, placed poorly: 0.5  Absent or unrecognisable: 0  Correct time:  Unit correct, placed properly: 2  Unit correct, placed poorly: 1  United distorted, incomplete but recognisable, placed properly: 1  Unit distorted, incomplete but recognisable, placed poorly: 0.5  Absent or unrecognisable: 0  Total: [ ]  *Not asked: 9* |
| **Memory - Registration** | |
| 204. This is John Brown. Try to remember his name. What is his name? | 201. This is John Brown. Try to remember his name. What is his name? |
| 205. He lives at: 42 West Street, Bedford. Where does he live? | 202. He lives at: 42 West Street, Bedford. Where does he live? |
| **Praxis - Ideomotor** | |
| 206. Show me how you wave goodbye | 203. Show me how you wave goodbye |
| 207. Show me how you would cut with scissors | 204. Imagine you are eating with a knife. Show me how you would cut with a knife. |
| 208. Show me how you would brush your teeth with a toothbrush | 205. Imagine you are holding a toothbrush. Show me how you would brush your teeth with a toothbrush |
| **Praxis - Ideational** | |
| 209. I am going to give you a piece of paper. When I do, take the paper in your right hand. Fold the paper in half with both hands, and put the paper down on your lap. | Removed – Brief Praxis Test as optional |
| 210. Put the paper in the envelope and seal the envelope. | Removed – Brief Praxis Test as optional |
| **Memory – Intentional Learning** | |
| 211. What was this man’s name? | 210. What was this man’s name? |
| 212. What was his address? | 211. What was his address? |
| **Abstract Thinking** | |
| 213. In what way are an apple and banana alike? | 208. In what way are an apple and banana alike? |
| 214. In what way are a shirt and dress alike? | 209. In what way are a shirt and dress alike? |
| 215. In what way are a table and a chair alike? | Removed |
|  | 206. In what way are red and yellow alike? |
|  | 207. In what way are a guitar and drums alike? |
| **Visual Perception** | |
| 216. Who is this?   - Queen - Pope, Archbishop, Bishop | Removed |
| 217. These pictures are taken from unusual angles. Can you tell me what they are?   - Spectacles - Shoe - Purse/suitcase - Cup and saucer - Telephone - Pipe | Removed |
|  | 212. Here you can see a drawing. This drawing is made up of 5 different images. Please point to each of these images that you can see in the drawing at the top of the page.   - Pear - Apple - Banana - Strawberry - Cherry |

**Table S2. Participants demographics by site**

|  |  | **Spain –Barcelona** | **U.K. –Cambridge** | **Ireland – Dublin** | **Greece** | **U.K. – London** | **Germany – Munich** | **Norway** | **France – Paris** |
| --- | --- | --- | --- | --- | --- | --- | --- | --- | --- |
| *n* = 223 |  | 26 | 7 | 33 | 49 | 36 | 31 | 21 | 20 |
| Mean age at assessment (SD) |  | 44.42 (9.72) | 42.00 (3.79) | 33.97 (6.22) | 33.04 (10.02) | 45.25 (10.86) | 40.61 (12.13) | 45.10 (12.75) | 46.85 (10.74) |
| Age group | Younger aged <40 | 9 (34.6) | 2 (28.6) | 28 (84.8) | 38 (77.6) | 14 (38.9) | 18 (58.1) | 7 (33.3) | 7 (35.0) |
|  | Older aged >40 | 17 (65.4) | 5 (71.4) | 5 (15.2) | 11 (22.4) | 22 (61.1) | 13 (41.9) | 14 (66.7) | 13 (65.0) |
| Sex | Female | 12 (46.2) | 3 (42.9) | 14 (42.4) | 22 (44.9) | 19 (52.8) | 12 (38.7) | 11 (52.4) | 12 (60.0) |
|  | Male | 14 (53.8) | 4 (57.1) | 19 (57.6) | 27 (55.1) | 17 (47.2) | 19 (61.3) | 10 (47.6) | 8 (40.0) |
| Level of intellectual disability | Mild | 16 (61.5) | 3 (42.9) | 17 (51.5) | 19 (38.8) | 14 (38.9) | 17 (54.8) | 7 ( 33.3) | 7 (35.0) |
|  | Moderate | 10 (38.5) | 4 (57.1) | 16 (48.5) | 25 (51.0) | 19 (52.8) | 14 (45.2) | 11 (52.4) | 13 (65.0) |
|  | Severe | 0 (0.0) | 0 (0.0) | 0 (0.0) | 5 (10.2) | 3 (8.3) | 0 (0.0) | 3 (14.3) | 0 (0.0) |
| Mean age of AD symptoms (SD) |  | 50.08 (6.25) | 30.00 (NA) | - | 40.60 (1.95) | 50.50 (4.36) | 56.33 (4.04) | - | 47.00 (NA) |
| Mean age of AD diagnosis (SD) |  | 52.00 (5.69) | 30.00 (NA) | - | 44.00 (4.30) | 54.83 (4.45) | 59.00 (3.00) | 47.00 (1.41) | 53.00 (5.66) |

| **Site** | **Local Ethics Approval** |
| --- | --- |
| Spain-Barcelona | Sant Pau Research Ethics Committee (IIBSP-DOW-2022-65) |
| U.K.-Cambridge | Wales Research Ethics Committee5 (21/WA/0365) |
| Ireland-Dublin | Faculty of Health Sciences Research Ethics Committee, Trinity College Dublin (2020606) |
| U.K.-London | North-West Wales Research Ethics Committee (13/WA/0194) |
| Germany-Munich | Ethics committee of the LMU medical faculty (17-126; 19-022) |
| Norway | Regional Committee for Medical Research Ethics South East Norway (2019/812) |
| France-Paris | Comité de Protection des Personnes Sud Méditéranée 2 |
| Greece | Greek Association of Alzheimer's Disease and  Related Disorders (Alzheimer Hellas) (82/19-10-2022) |

**Table S3. Local ethics approvals by site**

**Figure S1. ROC curve analysis of CAMCOG-DS-II classifying AD from cognitively stable participants**

**Table S4. AUC for CAMCOG-DS-II**

| **CAMCOG-DS-II** | **AUC (95% CI)** |
| --- | --- |
| Orientation | 0.75 (0.65–0.86) |
| Language | 0.69 (0.58–0.80) |
| Memory-New Learning | 0.76 (0.68–0.84) |
| Praxis | 0.68 (0.57–0.79) |
| Perception | 0.70 (0.60–0.80) |
| Executive function | 0.73 (0.63–0.83) |
| Total score | 0.75 (0.66–0.84) |

**Figure S2. ROC curve analysis of comparator cognitive tests classifying AD from cognitively stable participants**

**Table S5. AUC for comparator cognitive tests**

| **Test** | **AUC (95% CI)** |
| --- | --- |
| PAL first trial memory score | 0.79 (0.70–0.88) |
| Purdue Pegboard total score | 0.69 (0.56–0.83) |
| mCRT total immediate recall score | 0.82 (0.72–0.92) |
| mCRT total delayed recall score | 0.80 (0.69–0.90) |

**Table S6. Age group comparisons with demographics and performance on the CAMCOG-DS-II and comparator cognitive measures**

|  |  | **Younger aged <40** | **Older aged >40** | ***P* value** |
| --- | --- | --- | --- | --- |
| n |  | 123 | 100 |  |
| Age at assessment (mean (SD)) | | 31.49 (5.71) | 50.88 (6.64) | <.001 |
| Level of intellectual disability (%) | Mild | 63 (51.2) | 37 (37.0) | .047 |
|  | Moderate or severe | 60 (48.8) | 63 (63.0) |  |
| Site (%) | Barcelona | 9 (7.3) | 17 (17.0) | <.001 |
|  | Cambridge | 2 (1.6) | 5 (5.0) |  |
|  | Dublin | 28 (22.8) | 5 (5.0) |  |
|  | Greece | 38 (30.9) | 11 (11.0) |  |
|  | London | 14 (11.4) | 22 (22.0) |  |
|  | Munich | 18 (14.6) | 13 (13.0) |  |
|  | Norway | 7 (5.7) | 14 (14.0) |  |
|  | Paris | 7 (5.7) | 13 (13.0) |  |
| Sex (%) | Female | 59 (48.0) | 46 (46.0) | .875 |
|  | Male | 64 (52.0) | 54 (54.0) |  |
| AD diagnosis (%) | No | 122 (99.2) | 75 (75.0) | <.001 |
|  | Yes | 1 (0.8) | 25 (25.0) |  |
| Age at AD diagnosis (mean (SD)) | | 40.00 (NA) | 51.17 (7.45) | - |
| Age at AD symptoms (mean (SD)) | | 38.00 (NA) | 48.08 (7.67) | - |
| **Orientation** | | | | |
| Total score (median [IQR]) | | 11.00 [8.00, 12.00] | 8.00 [4.00, 11.00] | <.001 |
| **Language** | | | | |
| Comprehension (median [IQR]) | | 7.00 [5.00, 8.00] | 6.00 [4.00, 8.00] | .003 |
| Expression (median [IQR]) | | 12.50 [10.25, 14.00] | 12.00 [9.00, 13.00] | .002 |
| Total score (median [IQR]) | | 20.00 [16.00, 22.00] | 17.00 [12.00, 20.00] | .001 |
| **Memory-New learning** | | | | |
| Total score (median [IQR]) | | 14.00 [10.00, 16.00] | 8.00 [5.00, 13.00] | <.001 |
| **Praxis** | | | | |
| Actions to command (median [IQR]) | | 4.00 [3.00, 5.00] | 4.00 [2.00, 5.00] | .002 |
| Drawing/copying (median [IQR]) | | 7.00 [4.50, 9.00] | 5.00 [3.00, 7.25] | .001 |
| Total score (median [IQR]) | | 11.50 [8.50, 13.50] | 9.00 [5.00, 12.00] | <.001 |
| **Perception** | | | | |
| Total score (median [IQR]) | | 5.00 [4.00, 5.00] | 4.00 [2.00, 5.00] | <.001 |
| **Executive function** | | | | |
| Verbal fluency (median [IQR]) | | 3.00 [2.00, 3.00] | 2.00 [1.00, 3.00] | <.001 |
| Prospective memory (median [IQR]) | | 2.00 [1.00, 3.00] | 1.00 [0.00, 3.00] | <.001 |
| Inhibition (median [IQR]) | | 4.00 [3.00, 5.00] | 3.00 [0.00, 5.00] | <.001 |
| Attention (median [IQR]) | | 6.00 [5.00, 7.00] | 5.00 [2.00, 6.00] | <.001 |
| Abstract thinking (median [IQR]) | | 3.00 [0.00, 5.00] | 1.00 [0.00, 3.00] | <.001 |
| Total score (median [IQR]) | | 18.00 [14.00, 22.00] | 12.00 [6.00, 18.00] | <.001 |
| **CAMCOG total score** (median [IQR]) | | 75.50 [63.00, 86.50] | 56.50 [36.00, 77.00] | <.001 |
| **CANTAB PAL** | | | | |
| PAL first trial memory score (median [IQR]) | | 12.00 [8.00, 15.00] | 7.00 [2.75, 11.00] | <.001 |
| **mCRT** | | | | |
| Total immediate recall score (median [IQR]) | | 34.00 [30.00, 36.00] | 32.00 [23.50, 35.00] | .002 |
| Total delayed recall score (median [IQR]) | | 12.00 [10.00, 12.00] | 10.00 [4.75, 12.00] | .006 |
| Intrusions immediate recall (median [IQR]) | | 1.00 [0.00, 3.00] | 3.00 [0.50, 8.00] | <.001 |
| Intrusions delayed recall (median [IQR]) | | 0.00 [0.00, 1.00] | 1.50 [0.00, 3.00] | <.001 |
| **Purdue Pegboard** | | | | |
| Total score (median [IQR]) | | 25.50 [20.50, 35.25] | 16.50 [11.25, 20.75] | <.001 |

**Table S7. Sex comparisons with demographics and performance on the CAMCOG-DS-II and comparator cognitive measures**

|  |  | **Female** | **Male** | ***P* value** |
| --- | --- | --- | --- | --- |
| n |  | 105 | 118 |  |
| Age at assessment (mean (SD)) |  | 40.21 (11.34) | 40.16 (11.59) | .975 |
| Age Group (%) | Younger aged <40 | 59 (56.2) | 64 (54.2) | .875 |
|  | Older aged >40 | 46 (43.8) | 54 (45.8) |  |
| Level of intellectual disability (%) | Mild | 55 (52.4) | 45 (38.1) | .045 |
|  | Moderate or severe | 50 (47.6) | 73 (61.9) |  |
| Site (%) | Barcelona | 12 (11.4) | 14 (11.9) | .85 |
|  | Cambridge | 3 (2.9) | 4 (3.4) |  |
|  | Dublin | 14 (13.3) | 19 (16.1) |  |
|  | Greece | 22 (21.0) | 27 (22.9) |  |
|  | London | 19 (18.1) | 17 (14.4) |  |
|  | Munich | 12 (11.4) | 19 (16.1) |  |
|  | Norway | 11 (10.5) | 10 (8.5) |  |
|  | Paris | 12 (11.4) | 8 (6.8) |  |
| Sex (%) | Female | 105 (100.0) | 0 (0.0) | <.001 |
|  | Male | 0 (0.0) | 118 (100.0) |  |
| AD diagnosis (%) | No | 90 (85.7) | 107 (90.7) | 0.345 |
|  | Yes | 15 (14.3) | 11 (9.3) |  |
| Age at AD diagnosis (mean (SD)) | | 49.64 (7.45) | 52.09 (7.99) | 0.438 |
| Age at AD symptoms (mean (SD)) | | 46.41 (7.21) | 49.00 (8.67) | 0.475 |
| **Orientation** | | | | |
| Total score (median [IQR]) | | 10.00 [5.00, 12.00] | 10.00 [5.00, 12.00] | 0.647 |
| **Language** | | | | |
| Comprehension (median [IQR]) | | 7.00 [5.00, 8.00] | 6.00 [4.00, 8.00] | 0.07 |
| Expression (median [IQR]) | | 12.00 [10.00, 13.25] | 12.00 [9.00, 13.00] | 0.717 |
| Total score (median [IQR]) | | 19.00 [15.75, 21.00] | 18.00 [13.00, 21.00] | 0.267 |
| **Memory-New learning** | | | | |
| Total score (median [IQR]) | | 13.00 [7.00, 15.25] | 12.00 [7.00, 15.00] | 0.5 |
| **Praxis** | | | | |
| Actions to command (median [IQR]) | | 4.00 [3.00, 5.00] | 4.00 [3.00, 5.00] | 0.173 |
| Drawing/copying (median [IQR]) | | 6.25 [3.50, 9.00] | 6.00 [3.00, 8.00] | 0.477 |
| Total score (median [IQR]) | | 11.00 [7.00, 13.50] | 10.00 [6.00, 13.00] | 0.288 |
| **Perception** | | | | |
| Total score (median [IQR]) | | 4.00 [3.00, 5.00] | 4.00 [3.00, 5.00] | 0.37 |
| **Executive function** | | | | |
| Verbal fluency (median [IQR]) | | 2.00 [2.00, 3.00] | 2.00 [2.00, 3.00] | 0.631 |
| Prospective memory (median [IQR]) | | 2.00 [1.00, 3.00] | 2.00 [0.00, 3.00] | 0.205 |
| Inhibition (median [IQR]) | | 4.00 [2.00, 5.00] | 4.00 [1.00, 5.00] | 0.459 |
| Attention (median [IQR]) | | 5.00 [4.00, 6.25] | 6.00 [4.00, 7.00] | 0.472 |
| Abstract thinking (median [IQR]) | | 3.00 [0.00, 5.25] | 2.00 [0.00, 4.00] | 0.028 |
| Total score (median [IQR]) | | 16.00 [10.75, 21.25] | 15.00 [9.00, 20.00] | 0.271 |
| **CAMCOG total score** (median [IQR]) | | 72.00 [51.25, 83.25] | 66.25 [46.00, 82.25] | 0.225 |
| **CANTAB PAL** | | | | |
| PAL first trial memory score (median [IQR]) | | 10.00 [4.00, 13.00] | 10.00 [6.00, 13.00] | 0.611 |
| **mCRT** | | | | |
| Total immediate recall score (median [IQR]) | | 34.00 [29.00, 36.00] | 33.00 [28.00, 35.75] | 0.723 |
| Total delayed recall score (median [IQR]) | | 11.00 [9.00, 12.00] | 11.00 [8.50, 12.00] | 0.635 |
| Intrusions immediate recall (median [IQR]) | | 2.00 [0.00, 5.00] | 2.00 [0.00, 4.00] | 0.951 |
| Intrusions delayed recall (median [IQR]) | | 1.00 [0.00, 2.25] | 0.00 [0.00, 2.00] | 0.613 |
| **Purdue Pegboard** | | | | |
| Total score (median [IQR]) | | 21.00 [16.00, 26.00] | 19.00 [13.50, 33.50] | 0.649 |

**Table S8. Intellectual disability level group comparisons with demographics and performance on the CAMCOG-DS-II and comparator cognitive measures**

| **Level of Intellectual Disability** |  | **Mild** | **Moderate or Severe** | ***P* value** |
| --- | --- | --- | --- | --- |
| n |  | 100 | 123 |  |
| Age at assessment (mean (SD)) | | 39.24 (10.97) | 40.95 (11.81) | .268 |
| Age Group (%) | Younger aged <40 | 63 (63.0) | 60 (48.8) | .047 |
|  | Older aged >40 | 37 (37.0) | 63 (51.2) |  |
| Level of intellectual disability (%) | Mild | 100 (100.0) | 0 (0.0) | <.001 |
|  | Moderate or severe | 0 (0.0) | 123 (100.0) |  |
| Site (%) | Barcelona | 16 (16.0) | 10 (8.1) | .338 |
|  | Cambridge | 3 (3.0) | 4 (3.3) |  |
|  | Dublin | 17 (17.0) | 16 (13.0) |  |
|  | Greece | 19 (19.0) | 30 (24.4) |  |
|  | London | 14 (14.0) | 22 (17.9) |  |
|  | Munich | 17 (17.0) | 14 (11.4) |  |
|  | Norway | 7 (7.0) | 14 (11.4) |  |
|  | Paris | 7 (7.0) | 13 (10.6) |  |
| Sex (%) | Female | 55 (55.0) | 50 (40.7) | .045 |
|  | Male | 45 (45.0) | 73 (59.3) |  |
| AD diagnosis (%) | No | 94 (94.0) | 103 (83.7) | .03 |
|  | Yes | 6 (6.0) | 20 (16.3) |  |
| Age at AD diagnosis (mean (SD)) | | 52.17 (5.56) | 50.26 (8.25) | .605 |
| Age at AD symptoms (mean (SD)) | | 50.08 (6.25) | 46.50 (8.35) | .36 |
| **Orientation** | | | | |
| Total score (median [IQR]) | | 11.00 [9.00, 12.00] | 8.00 [4.00, 11.00] | <.001 |
| **Language** | | | | |
| Comprehension (median [IQR]) | | 8.00 [6.00, 8.00] | 6.00 [3.25, 7.00] | <.001 |
| Expression (median [IQR]) | | 13.00 [12.00, 14.00] | 11.00 [8.00, 12.75] | <.001 |
| Total score (median [IQR]) | | 21.00 [18.00, 22.00] | 16.00 [12.00, 19.75] | <.001 |
| **Memory-New learning** | | | | |
| Total score (median [IQR]) | | 14.00 [10.00, 16.50] | 9.00 [6.00, 14.00] | <.001 |
| **Praxis** | | | | |
| Actions to command (median [IQR]) | | 5.00 [4.00, 5.00] | 4.00 [2.00, 5.00] | <.001 |
| Drawing/copying (median [IQR]) | | 7.50 [5.50, 10.00] | 4.50 [2.50, 7.00] | <.001 |
| Total score (median [IQR]) | | 12.00 [9.00, 14.50] | 8.50 [5.00, 11.00] | <.001 |
| **Perception** | | | | |
| Total score (median [IQR]) | | 4.50 [4.00, 5.00] | 4.00 [2.00, 5.00] | <.001 |
| **Executive** | | | | |
| Verbal fluency (median [IQR]) | | 3.00 [2.00, 4.00] | 2.00 [1.00, 3.00] | <.001 |
| Prospective memory (median [IQR]) | | 2.50 [1.00, 3.00] | 2.00 [0.00, 3.00] | <.001 |
| Inhibition (median [IQR]) | | 4.00 [3.00, 6.00] | 3.00 [0.00, 5.00] | <.001 |
| Attention (median [IQR]) | | 6.00 [5.00, 7.00] | 5.00 [3.00, 6.00] | <.001 |
| Abstract thinking (median [IQR]) | | 4.00 [1.00, 6.00] | 1.00 [0.00, 3.00] | <.001 |
| Total score (median [IQR]) | | 19.00 [15.00, 23.00] | 12.00 [7.00, 18.00] | <.001 |
| **CAMCOG total score** (median [IQR]) | | 82.00 [68.25, 91.75] | 61.50 [39.00, 75.00] | <.001 |
| **CANTAB PAL** | | | | |
| PAL first trial memory score (median [IQR]) | | 9.00 [5.00, 13.00] | 10.00 [3.00, 13.00] | .418 |
| **mCRT** | | | | |
| Total immediate recall score (median [IQR]) | | 35.00 [32.00, 36.00] | 31.50 [26.00, 35.00] | <.001 |
| Total delayed recall score (median [IQR]) | | 12.00 [10.00, 12.00] | 10.00 [6.00, 12.00] | <.001 |
| Intrusions immediate recall (median [IQR]) | | 1.50 [0.00, 4.00] | 2.00 [0.00, 6.00] | .433 |
| Intrusions delayed recall (median [IQR]) | | 0.50 [0.00, 2.00] | 1.00 [0.00, 2.00] | .77 |
| **Purdue Pegboard** | | | | |
| Total score (median [IQR]) | | 22.50 [19.25, 31.00] | 17.00 [12.75, 31.50] | .024 |
